# A comparative study of PCS and PAM50 prostate cancer classification schemes

**DOI:** 10.1038/s41391-021-00325-4

**Published:** 2021-02-02

**Authors:** Junhee Yoon, Minhyung Kim, Edwin M. Posadas, Stephen J. Freedland, Yang Liu, Elai Davicioni, Robert B. Den, Bruce J. Trock, R. Jeffrey Karnes, Eric A. Klein, Michael R. Freeman, Sungyong You

**Affiliations:** 1 Department of Surgery, Cedars-Sinai Medical Center, Los Angeles, CA USA; 2 Urologic Oncology Program & Uro-Oncology Research Program, Cedars-Sinai Cancer, Cedars-Sinai Medical Center, Los Angeles, CA USA; 3 Division of Oncology, Department of Medicine, Cedars-Sinai Medical Center, Los Angeles, CA USA; 4 Division of Urology, Department of Surgery, Veteran Affairs Healthcare System, Durham, NC USA; 5 Decipher Biosciences Inc., San Diego, CA USA; 6 Department of Radiation Oncology, Jefferson Medical College of Thomas Jefferson University, Philadelphia, PA USA; 7 James Buchanan Brady Urological Institute, Johns Hopkins Hospital, Baltimore, MD USA; 8 Department of Urology, Mayo Clinic, Rochester, MN USA; 9 Glickman Urological and Kidney Institute, Cleveland Clinic, Cleveland, OH USA; 10 Department of Biomedical Sciences, Cedars-Sinai Medical Center, Los Angeles, CA USA; 11 Department of Medicine, University of California, Los Angeles, CA USA

**Keywords:** Prostate cancer, Prognostic markers

## Abstract

**Background:**

Two prostate cancer (PC) classification methods based on transcriptome profiles, a de novo method referred to as the “Prostate Cancer Classification System” (PCS) and a variation of the established PAM50 breast cancer algorithm, were recently proposed. Both studies concluded that most human PC can be assigned to one of three tumor subtypes, two categorized as luminal and one as basal, suggesting the two methods reflect consistency in underlying biology. Despite the similarity, differences and commonalities between the two classification methods have not yet been reported.

**Methods:**

Here, we describe a comparison of the PCS and PAM50 classification systems. PCS and PAM50 signatures consisting of 37 (PCS37) and 50 genes, respectively, were used to categorize 9,947 PC patients into PCS and PAM50 classes. Enrichment of hallmark gene sets and luminal and basal marker gene expression were assessed in the same datasets. Finally, survival analysis was performed to compare PCS and PAM50 subtypes in terms of clinical outcomes.

**Results:**

PCS and PAM50 subtypes show clear differential expression of PCS37 and PAM50 genes. While only three genes are shared in common between the two systems, there is some consensus between three subtype pairs (PCS1 versus Luminal B, PCS2 versus Luminal A, and PCS3 versus Basal) with respect to gene expression, cellular processes, and clinical outcomes. PCS categories displayed better separation of cellular processes and luminal and basal marker gene expression compared to PAM50. Although both PCS1 and Luminal B tumors exhibited the worst clinical outcomes, outcomes between aggressive and less aggressive subtypes were better defined in the PCS system, based on larger hazard ratios observed.

**Conclusion:**

The PCS and PAM50 classification systems are similar in terms of molecular profiles and clinical outcomes. However, the PCS system exhibits greater separation in multiple clinical outcomes and provides better separation of prostate luminal and basal characteristics.

## Introduction

There has been much recent progress in the use of prostate cancer (PC) genomics to identify drivers of aggressive disease. These advances have spurred development of commercially available genomic classifiers that can identify aggressive tumors at high risk of progression to metastatic and/or castration resistant PC (CRPC) [1–7]. Despite this technology, there is no universally accepted or widely used molecular subtyping system for PC, unlike other cancers such as breast cancer, in which luminal A, luminal B, and basal subclassification are commonplace and clinically meaningful. Despite these limitations, PC subtypes have been proposed based on genomic criteria, such as various somatic alterations in chromatin sequence, (e.g., TMPRSS-ERG fusions [8, 9]) and androgen receptor amplification [3, 4]. The Cancer Genome Atlas identified several genomic PC subtypes, referred to as ERG, ETV1, ETV4, FLI1, SPOP, FOXA1, IDH1, and Other [7]. Tomlins et al. [10] described four subtypes based on gene expression (ERG+, ETV+, SPINK1+, and Triple Negative (ERG−/ETS−/SPINK1−)). However, the clinical applicability of these PC genomic classifiers has been limited [11, 12].

Many reports have proposed gene expression signatures as a means of tumor classification [13–18]. Two transcriptome-based classification methods were recently reported that categorize PC into three subtypes [19, 20]. Our group described the prostate cancer classification system (PCS), an integrated approach employing activation signatures of 14 pathways associated with PC biology to interrogate a virtual cohort of 1321 clinical samples [19]. Two luminal subtypes (PCS1 and PCS2) and one basal subtype (PCS3) were described in this report. PCS1 tumors exhibited the poorest clinical outcomes, including increased risk of metastatic progression, PC-specific mortality, and overall survival. In contrast, no significant differences in clinical outcomes were observed between PCS2 and PCS3; however, both PCS1 and PCS3 tumors were enriched in bone metastases in comparison to PCS2 [21]. In the initial report, we validated the PCS scheme with ten independent patient cohorts and 19 laboratory models of PC. In line with this, PCS1-specific genes were highly expressed in androgen receptor signaling inhibitor (ARSI) resistant PC [19] and this was independently validated by our recent study using a novel circulating tumor cell RNA assay system [22]. The initial You et al. study [19] was the first to categorize PC into only three subtypes using genomic approaches.

Zhao et al. [20] applied a variation of the widely used PAM50 breast cancer classifier to a large PC transcriptome dataset. PAM50 classifies breast cancer into luminal A (LumA), luminal B (LumB), HER2, Normal-like, and Basal subgroups [23]. The adaptation of PAM50 to PC focused on luminal and basal PC phenotypes and disregarded the HER2 and Normal-like subtypes described in the standard PAM50 system. Similar to the PCS system, PAM50 revealed that transcriptome data alone can divide PCs into only three subtypes, LumA, LumB, and Basal. These subgroups were associated with varied clinical behaviors. LumB exhibited the worst survival rate, while LumA and Basal subtypes exhibited similar clinical outcomes with better survival rates. In comparison to other classification schemes, which identified as many as seven PC subtypes [1–7], it is intriguing that both PCS and PAM50 concluded that PCs can be categorized into two distinct luminal and one basal subtype using only transcriptome data.

To date, no comparison has been made between these two different three-category classification systems. To address this gap, here we present a comparison of the PCS and PAM50 systems. We applied the PCS and PAM50 methods to two large PC transcriptome datasets: (1) the Prostate Cancer Transcriptome Atlas (PCTA) and (2) the Decipher GRID^TM^ database (GRID) (Supplementary Table 1). The PCTA is a virtual cohort consisting of 1,321 PC transcriptome profiles. These data were used to develop the PCS [19]. The GRID is a cohort consisting of 8626 PC transcriptome profiles, a subset of which was employed in the Zhao et al. study [20]. We hypothesized that the PCS and PAM50 systems have many similarities as well as differences in terms of molecular profiles and clinical outcomes. We thus performed a comparative analysis assessing three different measures: (1) gene expression patterns, (2) pathway associations, and (3) correlation with clinical outcomes.

## Methods

### Statistical analysis

To examine the association between clinical outcomes and PCS and PAM50 categories in the tumors from the GRID dataset, we used Kaplan–Meier survival analysis and Cox proportional hazards regression analysis with the following outcomes: biochemical recurrence (BCR), metastasis (Met), and PC-specific mortality (PCSM). To test whether PCS or PAM50 classification was prognostic independent of other clinical variables, multivariable analyses were performed adjusting for pathological grade, which was categorized into three groups with Gleason sum score of less than 7, equal to 7, and more than 7. All analyses were conducted using Python (version 2.7). *p* < 0.05 was considered statistically significant. Detailed information of transcriptome datasets and analysis procedures used in this study are described in Supplementary Methods.

## Results

### Conserved gene expression patterns between PCS and PAM50

**Fig. 1:**
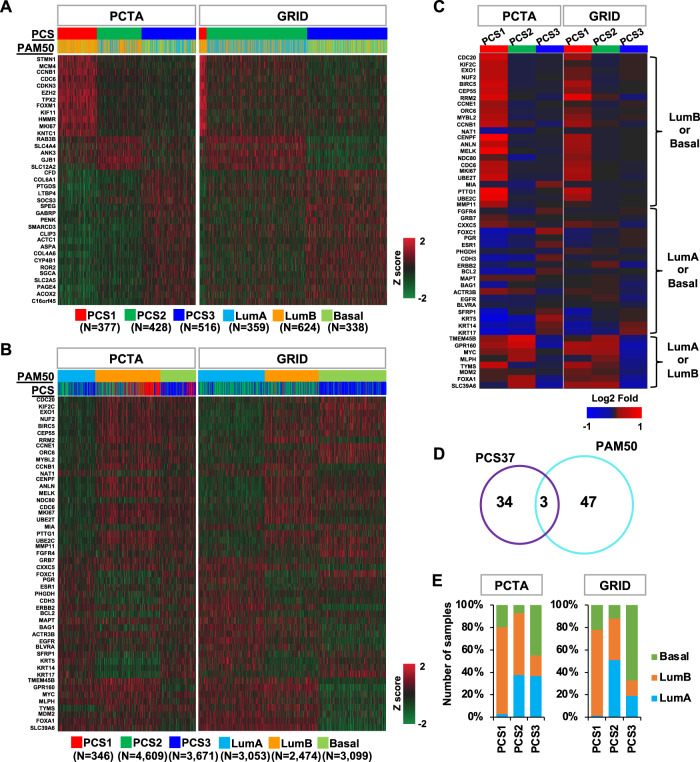
PCS37 and PAM50 genes in the PCTA and the GRID datasets. The heatmap depicts differential expression of PCS37 and PAM50 in the PCTA and GRID datasets based on **A** PCS and **B** PAM50 grouping. **C** The heatmap shows differential expression of PAM50 genes in the PCTA and GRID datasets based on PCS grouping. **D** The Venn diagram of PCS37 and PAM50. **E** The distribution of PAM50 subtypes across three PCS subtypes in the PCTA and GRID datasets. PCS prostate cancer subtype, LumA Luminal A subtype, LumB Luminal B subtype, Basal Basal subtype.

We applied the PCS and PAM50 classifiers to assign the tumors from the PCTA cohort (*n* = 1,321) [19] and the GRID cohort (*n* = 8,626) into PCS1-3 and LumA/B and Basal subtypes. Differential expression patterns of PCS37 genes [19] were displayed by PCS classes with the PCTA and the GRID datasets (Fig. 1A and Supplementary Fig. 1), showing distinct expression patterns of individual PCS class-specific genes. For example, 12 PCS1-specific genes (STMN1, MCM4, CCNB1, CDC6, CDKN3, EZH2, TPX2, FOXM1, KIF11, HMMR, MKI67, and KNTC1) are only highly expressed in PCS1 tumors (Fig. 1A). The GRID contains 4% (346/8,626) PCS1, the most aggressive PCS subtype, as most specimens were primary PCs. In the PCTA cohort, containing 260 metastatic PCs, 26% (377/1,321) of patients were assigned to PCS1. In total, 66% (176/260) of metastatic PCs belong to this PCS1 subtype. LumB, the most aggressive PAM50 subtype, accounts for 29% (2,474/8,626) of the GRID cohort and 47% (624/1,321) of the PCTA cohort. Of 260 metastatic PCs in the PCTA cohort, 170 (65%) patients were assigned LumB.

We then assessed PAM50 gene expression by PAM50 classes with both datasets (Fig. 1B). These 50 genes also showed distinct expression patterns among the classes. However, their expression was not unique to a specific subtype. For example, some genes that were highly expressed in LumB also showed high expression in Basal. In addition, some genes (e.g., NAT1, GRB7, MAPT) did not show a clear differential expression pattern among the PAM50 classes. Both PCS37 and PAM50 displayed clear separation into three PC classes. We further assessed the expression pattern of PAM50 genes by the PCS classifier (Fig. 1C). Of note, genes with high expression in LumB or Basal were highly expressed in PCS1. These genes exhibit mostly low expression in LumA.

Given that both classification methods resolve all PCs into three subtypes, it is interesting that only three genes (CCNB1, CDC6, and MKI67) overlap between the two systems (Fig. 1D). These three genes show high expression in both PCS1 and LumB. We observed all PCS categories in all classification groups as defined by PAM50 in both PCTA and GRID (Fig. 1E). We found a high frequency of PCS2 in LumA, but not PCS1 and PCS3. LumB was enriched for PCS1, while Basal subtype but not LumA or LumB was enriched for PCS3. This was validated by visualizing the distribution of the tumors from each category and their overlaps using PCS37 and PAM50 gene expression (Supplementary Fig. 2A). We then defined centroids of each categories and estimated the pairwise distances between the PCS and PAM50 categories (Supplementary Fig. 2B and Supplementary Table 2). Consistent with the enrichment of sample numbers shown in Fig. 1E, three subtype pairs (PCS1 versus LumB, PCS2 versus LumA, and PCS3 versus Basal) exhibit the shortest distance between centroids. Taken together, three subtype pairs (PCS1 versus LumB, PCS2 versus LumA, and PCS3 versus Basal) show comparable enrichment in terms of number of tumors commonly included in both subtypes, despite the small number of genes shared by PCS37 and PAM50.

### Pairwise overlap of PCS and PAM50 classes in cellular functions

**Fig. 2:**
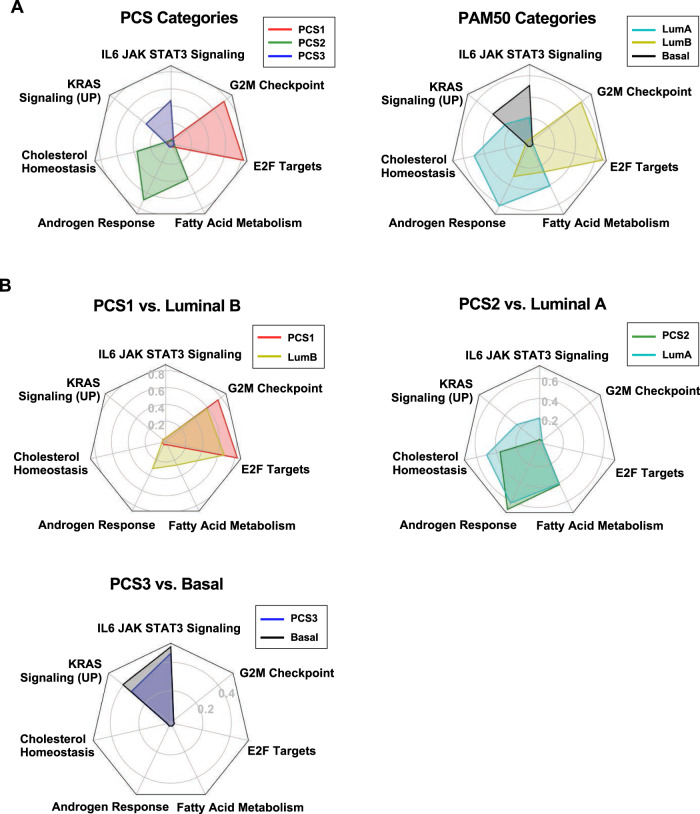
Enriched cellular processes of PCS and PAM50 subtypes in the PCTA. **A** Enriched seven hallmark gene sets of PCS (left) and PAM50 (right) categories were displayed with radar chart in the PCTA cohort. **B** Radar chart illustrates overlaps of enriched hallmark gene sets by PCS1 versus LumB, PCS2 versus LumA, and PCS3 versus Basal in the PCTA cohort. LumA Luminal A subtype, LumB Luminal B subtype, Basal Basal subtype.

We computed enrichment scores using the GSEA method [24] with hallmark gene sets [25] for all the PCS and PAM50 categories. In the PCTA, PCS1 samples were highly enriched with E2F targets and G2M checkpoint; PCS2 samples were enriched with androgen response, fatty acid metabolism, and cholesterol homeostasis; and PCS3 was enriched with IL6-JAK-STAT3 signaling and KRAS signaling (UP) (Fig. 2A). PAM50-classified samples in the PCTA showed an overall similar enrichment result with PCS classification (Fig. 2A). LumA samples were enriched in fatty acid metabolism, androgen response, cholesterol homeostasis. LumB samples were enriched with the hallmark gene sets of PCS1, such as E2F targets and G2M checkpoint. Basal samples were enriched in the same hallmark gene sets as PCS3 (Fig. 2B). In the GRID, PCS1, and PCS2 were enriched in E2F targets, G2M checkpoint, fatty acid metabolism, androgen response, and cholesterol homeostasis, but PCS3 did not show any significant enrichment of any of these hallmark gene sets. PAM50-classified samples in the GRID showed the same enrichment pattern. LumB exhibited higher ES of E2F targets and G2M checkpoint than LumA, but the highest score was androgen response in LumB-enriched hallmark gene sets (Supplementary Fig. 3A). The highest enrichment score in LumA was androgen response, similar to PCS2 (Supplementary Fig. 3B).

### Luminal and basal marker expression in PCS and PAM50 classifications

**Fig. 3:**
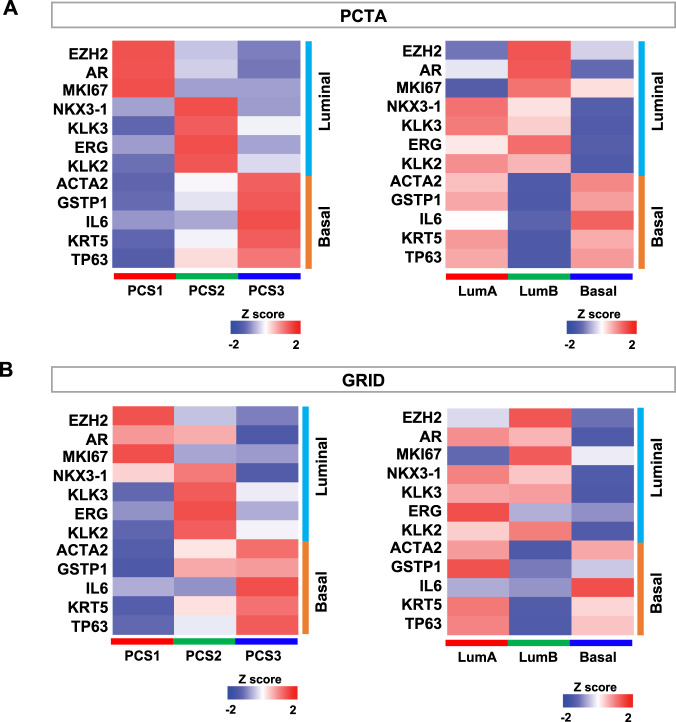
Gene expression of Luminal and Basal cell markers in the PCTA and the GRID. The heatmap displays expression of the Luminal and Basal cell marker genes in the PCTA (**A**) and the GRID (**B**).

We assessed expression levels of luminal and basal cell marker genes in the PCTA and the GRID datasets [19, 26]. Expression of these marker genes was highly concordant with PCS classification in both the PCTA (Fig. 3A) and the GRID (Fig. 3B), consistent with our prior results in the initial PCS report [19]. In the PAM50 classes, LumB and Basal subtypes display clear high and low expression of luminal and basal marker genes, respectively. However, LumA tumors exhibit mixed marker gene expression, indicating a composite luminal and basal expression phenotype. This result suggests that the PCS classification scheme is superior in separating prostatic luminal and basal gene expression.

### PCS1 and LumB are associated with the worst survival outcomes

**Fig. 4:**
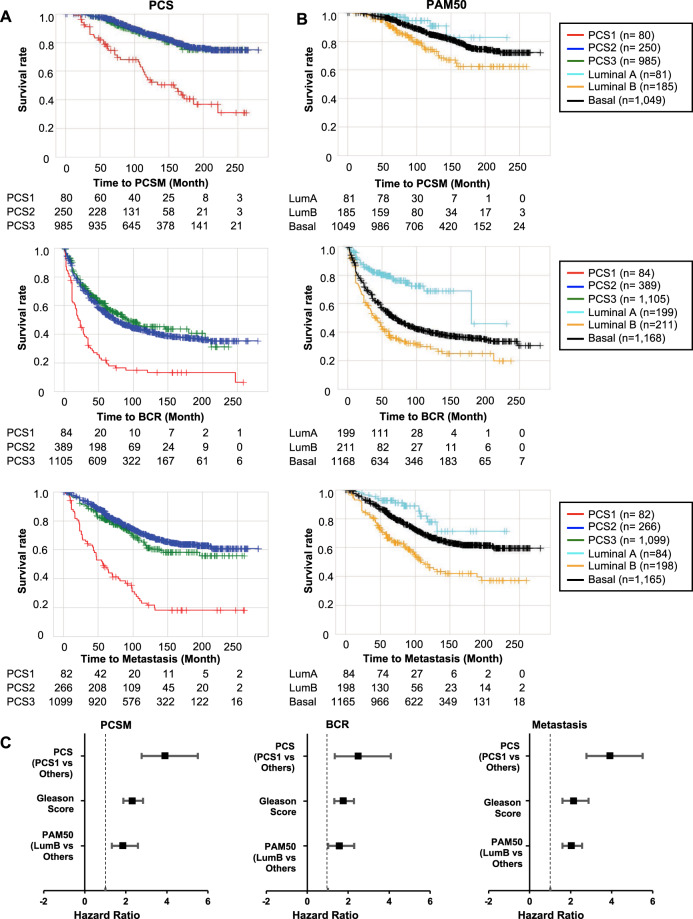
Clinical outcomes in distinct PCS and PAM50 subtypes in the GRID. Kaplan–Meier survival curves shows differential clinical outcome association of PCS (**A**) and PAM50 (**B**) categorization. Tables below the KM plot represent the number at risk. (PCSM prostate cancer-specific survival, BCR biochemical recurrence, Met metastasis.) **C** Forest plots display hazard ratios of PCS, PAM50, and Gleason grade against PCSM, BCR, and Met. (GS Gleason score).

We examined relationships between subtype category and the clinical outcomes of PCSM, BCR, and Met using the GRID. PCS1 within the PCS categories (Fig. 4A) and LumB within the PAM50 categories (Fig. 4B) were the most aggressive for all three metrics (PCSM, BCR, and Met). Cox proportional hazard analysis was performed using the two classification systems. In univariable analysis using the PCS scheme, the PCS1 subtype exhibited the highest hazard ratio (HR) (PCSM: 3.91 [2.78–5.52], BCR: 2.48 [1.34–4.08], Met: 3.91 [2.78–5.52]). Similarly, the LumB subtype exhibited the highest HR (PCSM: 1.85 [1.32–2.61], BCR: 1.54 [1.01–2.29], Met: 2.04 [1.58–2.55]) (Fig. 4C). In all combinations of pairwise comparisons, except for LumB compared to LumA in BCR (HR = 3.41 [2.40–4.87]), PCS1 exhibited higher HRs compared to LumB (Supplementary Table 3). We also performed multivariable analysis of PCS or PAM50 adjusting for Gleason score (Table 1). After the adjustment, PCS still showed larger HR (PCSM: 3.76 [2.17–6.25], BCR: 2.03 [1.39–2.96], Met: 3.38 [2.26–5.06]) compared to PAM50 (PCSM: 1.51 [0.96–2.38], BCR: 1.43 [1.14–1.79], Met: 1.73 [1.30–2.29]). Importantly, we found that controlling for Gleason score did not dramatically affect the HRs in either PCS or PAM50, indicating both are largely independent of Gleason grade for predicting patient outcomes. Of note, both multivariable PCS and PAM50 models with Gleason score exhibited the same concordance index at 0.75. We further checked the association of each subtype and Decipher score [27], which is designed to predict metastasis risk after radical prostatectomy. The Decipher score was significantly higher in PCS1 and LumB compared to other subtypes (Supplementary Fig. 4A, B). Multivariable analysis of Decipher score after adjustment of Gleason score was performed to compare with PCS and PAM50. Decipher score exhibited the highest HRs and significant *p* values in PCSM, BCR, and Met (Supplementary Fig. 4C).

**Table 1:** Multivariable Cox proportional hazard regression analysis for PCS and PAM50 classification with clinical parameters.

Multivariable analysis with PCS				Multivariable analysis with PAM50			
Variable	Hazard ratio	*p* value	C-index	Variable	Hazard ratio	*p* value	C-index
PCSM							
PCS	3.76 (2.17–6.52)	<0.001	0.75	PAM50	1.51 (0.96–2.38)	0.073	0.75
Gleason score	2.16 (1.74–2.66)	<0.001		Gleason score	2.25 (1.83–2.78)	<0.001	
BCR							
PCS	2.03 (1.39–2.96)	<0.001	0.66	PAM50	1.43 (1.14–1.79)	0.002	0.67
Gleason score	1.71 (1.55–1.88)	<0.001		Gleason score	1.69 (1.54–1.86)	<0.001	
Met							
PCS	3.38 (2.26–5.06)	<0.001	0.71	PAM50	1.73 (1.30–2.29)	<0.001	0.72
Gleason score	2.03 (1.79–2.31)	<0.001		Gleason score	2.04 (1.80–2.32)	<0.001	

Multivariate analysis of PCS or PAM50 classification system with Gleason score was performed.

## Discussion

In this study, we performed a comprehensive cross-comparison of subtype assignments obtained from the PCS and PAM50 classification methods. We found that three subtype pairs (PCS1 versus LumB, PCS2 versus LumA, and PCS3 versus Basal) show conserved gene expression patterns in the tumors included in both subtypes. Distance metrics of subtype centroids followed by visualization showed a similarity between these three pairs of subtypes. Despite the fact that only three genes overlap between the PCS37 and PAM50 signatures, these three subtype pairs exhibit very similar enrichment of cellular processes, as well as the distance of the centroids between the subtypes (e.g., PCS1 and LumB). This result suggests high concordance of the two subtypes in cellular functions and clinical phenotypes. Finally, a further comparison was made in clinical outcomes with PCSM, BCR, and Met, demonstrating that PCS1 and LumB subtypes are the most aggressive tumors with the worst survival outcomes in each categorization scheme.

The two classification methods likely reflect underlying luminal and basal biology as described in basic PC studies [19, 20]. PCS was originally designed as an unbiased classification scheme, with 14 PC relevant pathway activation signatures that revealed two luminal and one basal phenotype, while the PAM50 as applied to PC was biased toward luminal and basal phenotypes at the outset [20]. As such, it is interesting that two distinct PC categorization schemes converged to similar conclusions about the manner in which PCs can be grouped from transcriptome data alone. As shown in Fig. 3, both PCS and PAM50 exhibit differential expression of luminal and basal marker genes. However, PCS classes show consistent luminal and basal marker gene expression with clear separation between PCS1/2 and PCS3, respectively, while LumA tumors have high expression of basal marker genes, which are highly expressed in Basal tumors as well. This suggests that PCS classification more accurately identifies prostate luminal and basal phenotypes expressed within the cancer specimens.

The development of classification schema such as PCS and PAM50 in PC represents an important evolution in the field. Rather than considering an isolated biomarker or limited groups of biomarkers, these approaches focus on patterns of gene expression that ultimately relate to key biological drivers of the disease process. Thus far, these classifiers have shown the capacity to enhance clinical prediction and prognostication beyond conventional clinical criteria used in practice today. Emerging data with PAM50 and PCS point toward their potential utility in personalizing the approach to PC care, particularly with the use of systemic therapies such as ARSIs (e.g., abiraterone, enzalutamide, apalutamide, and darolutamide) and taxanes (e.g., docetaxel and cabazitaxel). Analysis of samples from the SPARTAN clinical trial revealed clinically important differences in behavior in response to apalutamide, based on PAM50 classification. In SPARTAN, PAM50 Basal patients seemed to have a greater absolute benefit from ADT and apalutamide, suggesting these patients may benefit from intensification with ARSIs [28]. Consistent with this, analysis of PCS1 and LumB specimens in the GRID from patients receiving ADT showed higher rates of PCSM, BCR, and Met compared to PCS2/3 or LumA/Basal (Supplementary Fig. 5). However, absence of information on ADT response in the GRID prohibits analysis of relative performance of the PCS and PAM50 classifications with respect to this clinical variable. While encouraging, prospective validations of these data are needed prior to more widespread deployment of these approaches. Studies are now underway that will provide important insights into this question, including the ongoing NRG-GU-006/BALANCE study (NCT03371719), which used PAM50 as a stratification variable, and has now finished accrual and is expected to yield results within the next 3 years.

While tissue-based classification will remain an important standard in PC, the clinical behavior of this disease makes obtaining tissue samples from patients with metastatic disease challenging. Given the potential importance of these tools in advanced PC/metastatic CRPC, additional means of deploying these technologies is crucial. Fortunately, given recent technical advances, it will become possible to conduct genomic classifications using liquid biopsies [22]. These blood-based tools will also require prospective validation. Liquid biopsies are included in ongoing studies, including NRG-GU-006/BALANCE. These initial studies will provide a foundational experience for use of PCS and PAM50 in clinical practice.

In order for these classification schemes to be useful in a clinical setting, it is critical that they offer actionable prognostic information. We observed a disparate percentage of subtype assignments within the study cohorts. The GRID consists of a prospective cohort (*n* = 7,000) and a retrospective cohort (*n* = 1,626) as shown in Supplementary Fig. 1. In the GRID, only 3.7% of the prospective cohort and 5% of the retrospective cohort are classified as PCS1, whereas the PCTA cohort contains 19% of primary tumors classified as PCS1. The likely explanation for this difference is that the GRID and the PCTA contain different proportions of high grade tumors or tumors with distinct metastatic potential. However, these three cohorts include a similar percentage of high risk patients (Gleason score > 7), and PCS1 tumors exhibit the worst prognosis in low grade tumors (Gleason score ≦ 7) based on our study [19]. This indicates that the PCS categorization is a variable independent variable of Gleason grade. On the other hand, the proportion of PCS1 (6% in our previous study [19]) and the incidence rate of metastases were similar, with about 3–6% of newly diagnosed PC cases with metastasis [29, 30]. The PCS1 assignment rate can therefore vary according to metastatic potential independent of pathological grade.

It is also well known that dataset composition and choices for reference construction affect subsequent subtype calling. Of note, standard PAM50 classification used in Zhao et al. and the present study is profoundly affected by the composition of the sample cohort used for reference construction. Zhao et al. conducted median centering to the GRID retrospective and prospective cohorts separately prior to applying the PAM50 algorithm. Thus, the percentage of PAM50 categories was assigned different median values from each cohort. In this study, we applied single median values based on the GRID, combining retrospective and prospective cohorts because the median value approaches the actual population mean as the sample number increases. In future studies, it will be necessary to introduce a method for improving classification robustness by applying appropriate reference construction [31].

PC is one of the malignancies most affected by genetic factors [32], and a range of genomic alterations and structural variations associated with the clinical outcomes have been described [3, 7, 33]. However, the present study is based only on transcriptome data, and did not consider genetic or structural changes associated with the cancers in the individual PCS and PAM50 categories. Thus, our report identified differences and similarities between the two classification schemes but did not make comparisons with other PC subtyping systems. In the future, comparative analysis with other classification methods using multi-omics data [3, 7, 34] will complement the work described here.

In conclusion, PCS and PAM50 present a new lens through which PC may be viewed. There are important similarities in these signatures despite obvious differences in origin and performance (based on existing datasets or samples). Prospective validation of these tools is underway and will help to clarify how they may be most effectively deployed in clinical practice.

## Supplementary information

Supplementary text

Supplementary materials
