# Supplementary material for: A comparative study of PCS and PAM50 prostate cancer classification schemes
Source: Prostate Cancer Prostatic Dis. 2021 Feb 2;24(3):733–42. doi: 10.1038/s41391-021-00325-4 (PMC8326303; doi:10.1038/s41391-021-00325-4)
Supplement: Supplementary file 1 — Supplementary text [file 41391_2021_325_MOESM1_ESM.docx]

**Supplementary Methods**

**Transcriptome datasets**

Two large transcriptome datasets were used to compare the PCS and PAM50 subclassification systems: the PCTA dataset and the Decipher Genomics Research Informatics Database (GRID^TM^, Decipher Biosciences, San Diego, CA) (**Supplementary Table 1**). The PCTA dataset, consisting of 1,321 PC transcriptome profiles for 18,390 genes^1^, was originally employed to develop the PCS classification method as a discovery cohort. Because the PCTA dataset is a virtual cohort assembled from diverse sources produced by microarray and RNA-seq platforms, it was normalized by median centering and quantile scaling (MCQ) in order to reduce batch effects and other biases based on platform difference. PCS classification method was validated with 10 independent patient cohorts and 19 laboratory models of prostate cancer based on the microarray and RNA-seq profiles with no specific biases from gene expression analysis platforms. The PCTA data is publicly accessible at <http://www.thepcta.org>. The GRID contains 8,626 PC transcriptome profiles for 17,226 genes.^2^ This dataset was used for validation of the PCS method, as well as the initial application of the PAM50 method to PC classification. The GRID dataset was generated from clinical use of the Decipher test, which involved transcriptome analysis of primary PC using Affymetrix Human Exon 1.0 ST Arrays and was normalized by the Single Channel Array Normalization (SCAN) method^3^. ComBat^4^ was applied to reduce batch biases. A subset of the GRID dataset was obtained from retrospective studies with comprehensive clinical variables, including PC specific mortality (PCSM), biochemical recurrence (BCR), and metastasis (Met)^1^. This retrospective cohort dataset was used to examine associations of PCS or PAM50 categories with clinical outcomes^1^.

**Assignment of tumors into PCS and PAM50 categories**

Prior to apply classification algorithms, SCAN normalized gene expression data was centered by median expression value of all the samples from GRID dataset. Because the median value gets closer to the actual population mean as the sample number increases. Practically, it is more stable considering the situation of adding samples of newly measured. MCQ method includes median centering step, thus the PCTA dataset was directly used for PCS and PAM50 assignment. To assign tumors into PAM50 categories, we applied the PAM50 algorithm from Zhao el al.^2^. Briefly, we selected three classes, LumA, LumB and Basal out of the original five classes^5^. A sample was assigned to one of the three categories with the highest correlation coefficient (Spearman’s rho) to the centroid of each category. Assignment to PCS categories was as described^1^. In our initial development of the PCS, we described a classifier of 37-gene panel (PCS37) that accurately assigns individual tumors to one of the three PCS subtypes. The PCS37 gene classifier was employed to calculate probabilities for each PCS category using a Random Forest algorithm. The tumors were assigned to the category with the largest probability score from the Random Forest classifier.

**Pairwise similarity between PCS and PAM50 categories**

We used a dimension reduction approach to the gene expression profiles to visualize how many tumors between the subtypes from different classifier are common and different. This allows us to show sample distributions in the reduced dimension defined by the genes in the classifier so that we can measure similarities quantitatively between the subtypes. The first two principal components were used to determine the x and y coordinates of the 2-dimensional space. We found that more than 75% of the variance from PAM50 genes or PCS37 genes are explained by the first two principal components. We then set the centroids of each category based on the tumors within the same categories using the kernel density estimation and computed pairwise distances between PCS and PAM50 centroids^6^. For example, we selected PCS1 and LumB tumors and projected these onto the 2-dimensional space using PCS37 gene expression. The centroids of PCS1 and LumB were defined and the distances between the PCS1 and LumB centroids was computed. In the same way, we computed all the distances between PCS and PAM50 centroids in the PCTA and the GRID.

**Gene set enrichment analysis**

We performed Gene Set Enrichment Analysis (GSEA)^7^ with the 50 hallmark gene sets in the Molecular Signature Database (MSigDB)^8^. We found that seven out of the 50 hallmark gene sets were uniquely enriched in each of the PCS categories or PAM50 categories. The seven hallmark gene sets are E2F Targets, G2M Checkpoint, Fatty Acid Metabolism, Androgen Response, Cholesterol Homeostasis, IL6 JAK STAT3 Signaling and KRAS Signaling. We then visualized differential enrichment of the gene sets using a radar chart.

**ADT response prediction**

The PCTA cohort and GRID cohort does not provide any information on ADT responsiveness. The GRID only has information on whether or not patients received ADT. Using this information and clinical outcomes, we predicted ADT response indirectly (**Supplementary Figure 5**). Among patients receiving ADT, PCS1 and LumB exhibit higher rates of PCSM, BCR, and Met than those assigned to PCS2/3 or LumA/Basal.

**Supplementary Table and Figure legends**

**Supplementary Figure 1. The proportion of subtypes from retrospective and prospective samples of GRID cohort.** (A) The proportion of PCS subtypes across PAM50 subtypes in the all, retrospective, and prospective samples of GRID cohort. (B) The proportion of PAM50 subtypes across PCS subtypes in the all, retrospective, and prospective samples of GRID cohort.

**Supplementary Figure 2. Distance between categories from PCS and PAM50.** (**A**) Principal component score plot displays distribution of individual tumors from the PCTA and the GRID cohorts. Tumors from different PCS or PAM50 categories displayed with different colors. (**B**) Projection of the PCTA tumors or the GRID tumors onto the first 2 principal components. Tumors from the individual categories from PCS and PAM50 were projected and the density tumor distributions between the 2 categories were visualized with contours. Euclidean distance is measured between 2 centroids of each category in the projection. Dots with different colors indicates different PCS or PAM50 categories. PCS=Prostate Cancer Subtype, LumA=Luminal A subtype, LumB=Luminal B subtype, Basal=Basal subtype.

**Supplementary Figure 3. Enriched cellular processes of PCS and PAM50 subtypes in the GRID.** Radar chart illustrates overlaps of enriched hallmark gene sets by PCS1 versus LumB, PCS2 versus LumA in the PCTA cohort. PCS3 and Basal did not have enriched gene sets in the GRID cohort. LumA=Luminal A subtype, LumB=Luminal B subtype.

**Supplementary Figure 4. Association of Decipher score and two PC classifier**. (**A-B**) Violin plot of display the distribution of Decipher score of three PCS subtypes (A) and three PAM50 subtypes (B). Forest plots present hazard ratios of PCS, PAM50, Gleason grade, and Decipher score against PCSM, BCR, and Met.

**Supplementary Figure 5. Association of ADT treatment and two PC classifier in GRID cohort.** (**A**) Percent stacked bar plot present the ratios of ADT treated and untreated patients in PCS1 and PCS2/3 subtypes. (**B**) Percent stacked bar plot present the ratios of ADT treated and untreated patients in LumB and LumA/Basal subtypes. (**C-D**) Percent stacked bar plot present the ratios of PCSM incidence in PCS1 and PCS2/3 subtypes (**C**) and LumB and LumA/Basal subtypes (**D**). (**E-F**) Percent stacked bar plot present the ratios of BCR incidence in PCS1 and PCS2/3 subtypes (**E**) and LumB and LumA/Basal subtypes (**F**). (**G-H**) Percent stacked bar plot present the ratios of Metastasis incidence in PCS1 and PCS2/3 subtypes (**G**) and LumB and LumA/Basal subtypes (**H**).

**Supplementary Table 1**. Clinical details of patients in the PCTA and the GRID cohort.

**Supplementary Table 2.** Centroid distance between PCS subtypes and PAM50 subtypes in the PCTA (A) and in the GRID (B).

**Supplementary Table 3**. All the possible combinations of pairwise comparisons in each classification system.

**Reference**

1. You S, Knudsen BS, Erho N, Alshalalfa M, Takhar M, Al-Deen Ashab H *et al*. Integrated Classification of Prostate Cancer Reveals a Novel Luminal Subtype with Poor Outcome. *Cancer Res* 2016; **76**(17)**:** 4948-4958.

2. Zhao SG, Chang SL, Erho N, Yu M, Lehrer J, Alshalalfa M *et al*. Associations of Luminal and Basal Subtyping of Prostate Cancer With Prognosis and Response to Androgen Deprivation Therapy. *JAMA Oncol* 2017; **3**(12)**:** 1663-1672.

3. Piccolo SR, Sun Y, Campbell JD, Lenburg ME, Bild AH, Johnson WE. A single-sample microarray normalization method to facilitate personalized-medicine workflows. *Genomics* 2012; **100**(6)**:** 337-344.

4. Johnson WE, Li C, Rabinovic A. Adjusting batch effects in microarray expression data using empirical Bayes methods. *Biostatistics* 2007; **8**(1)**:** 118-127.

5. Parker JS, Mullins M, Cheang MC, Leung S, Voduc D, Vickery T *et al*. Supervised risk predictor of breast cancer based on intrinsic subtypes. *J Clin Oncol* 2009; **27**(8)**:** 1160-1167.

6. Tibshirani R, Hastie T, Narasimhan B, Chu G. Diagnosis of multiple cancer types by shrunken centroids of gene expression. *Proc Natl Acad Sci U S A* 2002; **99**(10)**:** 6567-6572.

7. Subramanian A, Tamayo P, Mootha VK, Mukherjee S, Ebert BL, Gillette MA *et al*. Gene set enrichment analysis: a knowledge-based approach for interpreting genome-wide expression profiles. *Proc Natl Acad Sci U S A* 2005; **102**(43)**:** 15545-15550.

8. Liberzon A, Birger C, Thorvaldsdottir H, Ghandi M, Mesirov JP, Tamayo P. The Molecular Signatures Database (MSigDB) hallmark gene set collection. *Cell Syst* 2015; **1**(6)**:** 417-425.
