# Supplementary material for: A comparative study of PCS and PAM50 prostate cancer classification schemes
Source: Prostate Cancer Prostatic Dis. 2021 Feb 2;24(3):733–42. doi: 10.1038/s41391-021-00325-4 (PMC8326303; doi:10.1038/s41391-021-00325-4)
Supplement: Supplementary file 2 — Supplementary materials [file 41391_2021_325_MOESM2_ESM.pptx]

## Slide 1
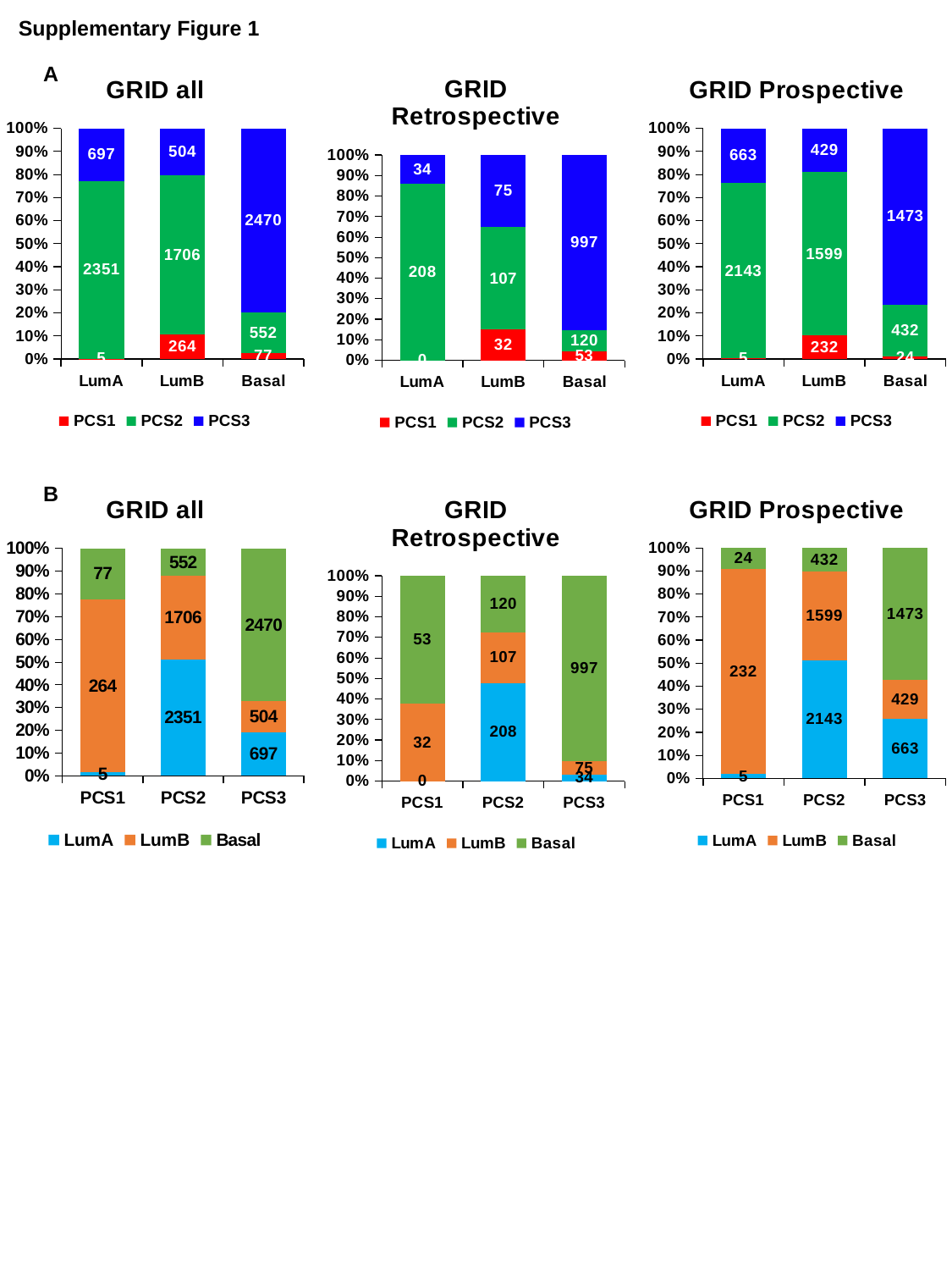

Supplementary Figure 1
### Chart: GRID Retrospective
| Category | PCS1 | PCS2 | PCS3 |
|---|---|---|---|
| LumA | 0.0 | 208.0 | 34.0 |
| LumB | 32.0 | 107.0 | 75.0 |
| Basal | 53.0 | 120.0 | 997.0 |
### Chart: GRID all
| Category | PCS1 | PCS2 | PCS3 |
|---|---|---|---|
| LumA | 5.0 | 2351.0 | 697.0 |
| LumB | 264.0 | 1706.0 | 504.0 |
| Basal | 77.0 | 552.0 | 2470.0 |A
### Chart: GRID Prospective
| Category | PCS1 | PCS2 | PCS3 |
|---|---|---|---|
| LumA | 5.0 | 2143.0 | 663.0 |
| LumB | 232.0 | 1599.0 | 429.0 |
| Basal | 24.0 | 432.0 | 1473.0 |
### Chart: GRID all
| Category | LumA | LumB | Basal |
|---|---|---|---|
| PCS1 | 5.0 | 264.0 | 77.0 |
| PCS2 | 2351.0 | 1706.0 | 552.0 |
| PCS3 | 697.0 | 504.0 | 2470.0 |B
### Chart: GRID Retrospective
| Category | LumA | LumB | Basal |
|---|---|---|---|
| PCS1 | 0.0 | 32.0 | 53.0 |
| PCS2 | 208.0 | 107.0 | 120.0 |
| PCS3 | 34.0 | 75.0 | 997.0 |
### Chart: GRID Prospective
| Category | LumA | LumB | Basal |
|---|---|---|---|
| PCS1 | 5.0 | 232.0 | 24.0 |
| PCS2 | 2143.0 | 1599.0 | 432.0 |
| PCS3 | 663.0 | 429.0 | 1473.0 |

## Slide 2
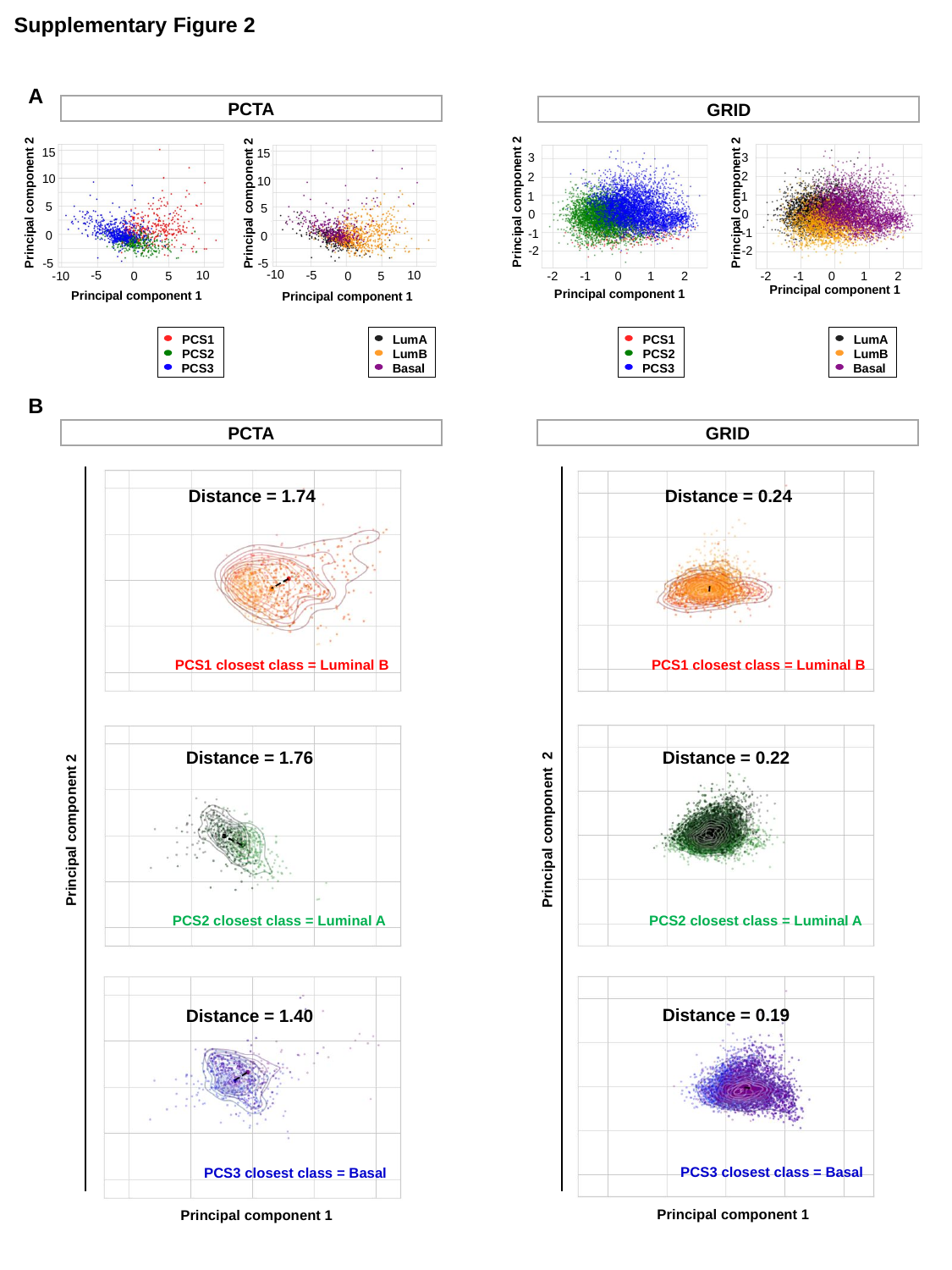

Supplementary Figure 2
A
PCTA
GRID
15
10
5
0
-5
-5
10
-10
0
5
15
10
5
0
-5
-10
-5
10
0
5
3
2
1
0
-1
-2
0
1
2
-2
-1
3
2
1
0
-1
-2
0
1
2
-2
-1
Principal component 2
Principal component 2
Principal component 2
Principal component 2
Principal component 1
Principal component 1
Principal component 1
Principal component 1
PCS1
PCS2
PCS3
LumA
LumB
Basal
PCS1
PCS2
PCS3
LumA
LumB
Basal
B
GRID
PCTA
Distance = 0.24
Distance = 1.74
PCS1 closest class = Luminal B
PCS1 closest class = Luminal B
Distance = 0.22
Distance = 1.76
Principal component 2
Principal component 2
PCS2 closest class = Luminal A
PCS2 closest class = Luminal A
Distance = 0.19
Distance = 1.40
PCS3 closest class = Basal
PCS3 closest class = Basal
Principal component 1
Principal component 1

## Slide 3
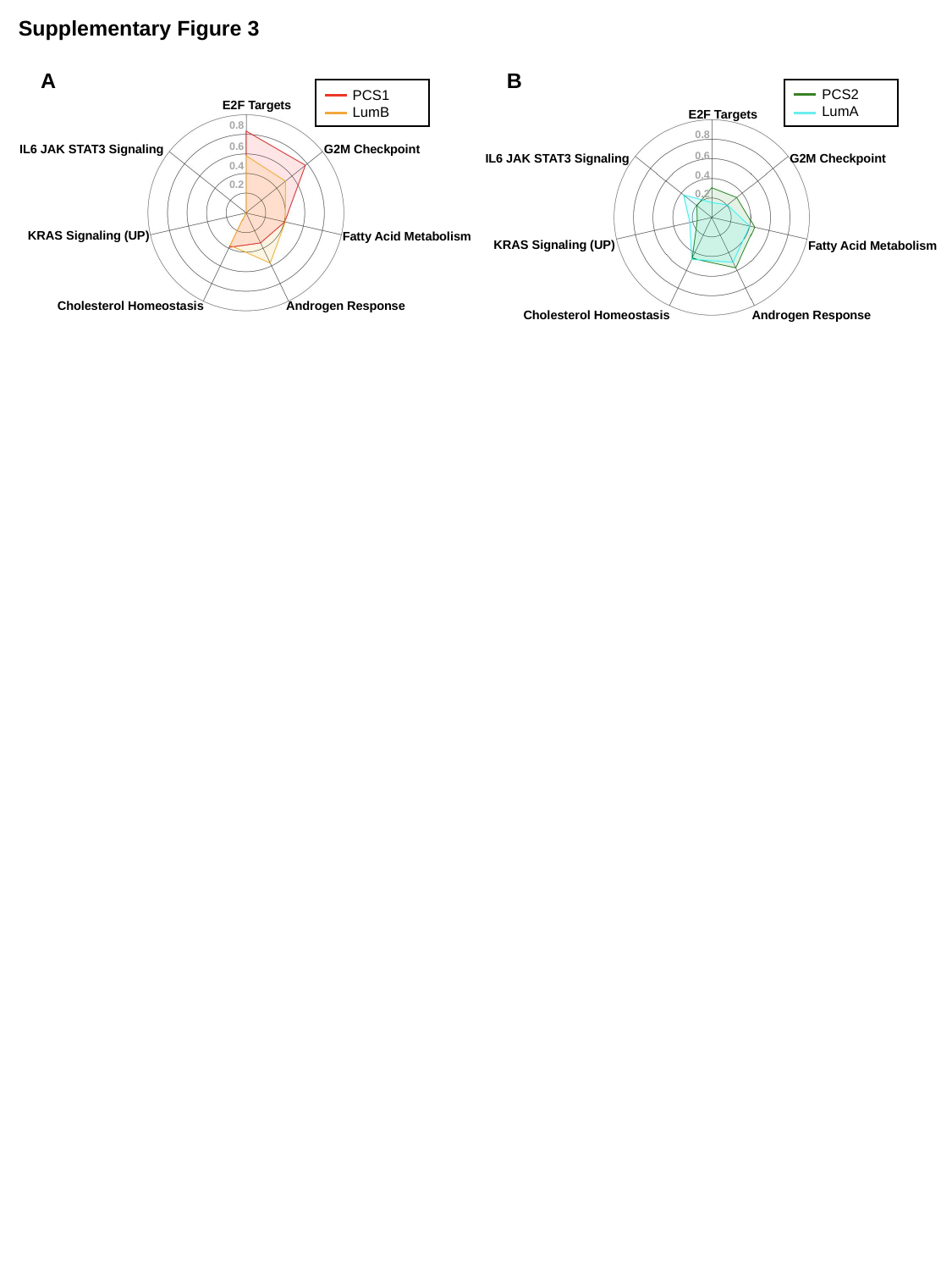

Supplementary Figure 3
A
B
PCS2
LumA
PCS1
LumB
E2F Targets
E2F Targets
0.8
0.8
0.6
IL6 JAK STAT3 Signaling
G2M Checkpoint
0.6
IL6 JAK STAT3 Signaling
G2M Checkpoint
0.4
0.4
0.2
0.2
KRAS Signaling (UP)
Fatty Acid Metabolism
KRAS Signaling (UP)
Fatty Acid Metabolism
Cholesterol Homeostasis
Androgen Response
Cholesterol Homeostasis
Androgen Response

## Slide 4
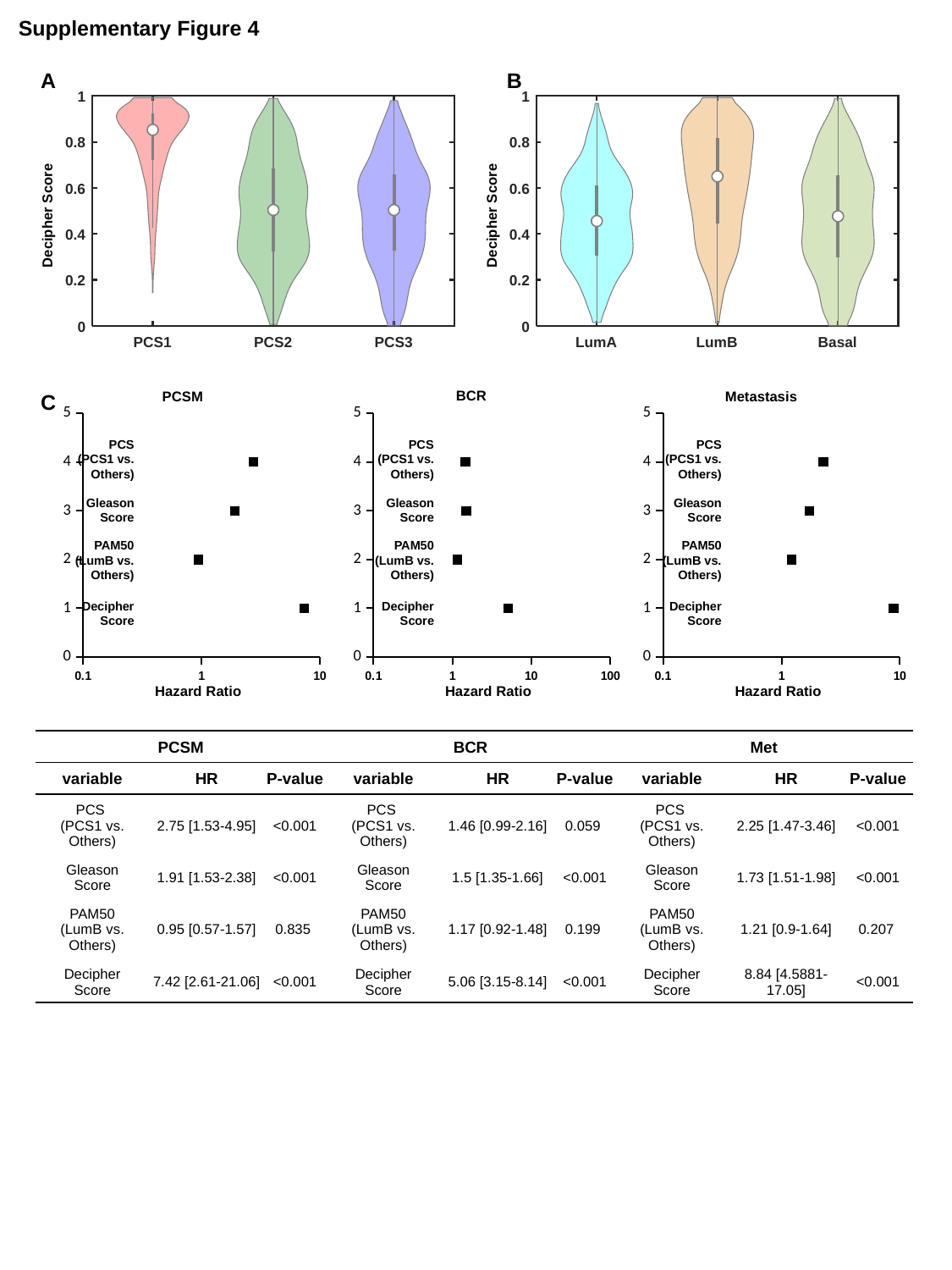

Supplementary Figure 4
A
B
Decipher Score
Decipher Score
BCR
Metastasis
PCSM
C
### Chart
| Category | |
|---|---|PCS
(PCS1 vs.
Others)
Gleason
Score
PAM50
(LumB vs.
Others)
Decipher
Score
Hazard Ratio
### Chart
| Category | |
|---|---|PCS
(PCS1 vs.
Others)
Gleason
Score
PAM50
(LumB vs.
Others)
Decipher
Score
Hazard Ratio
### Chart
| Category | |
|---|---|PCS
(PCS1 vs.
Others)
Gleason
Score
PAM50
(LumB vs.
Others)
Decipher
Score
Hazard Ratio
| PCSM | | | BCR | | | Met | | |
| --- | --- | --- | --- | --- | --- | --- | --- | --- |
| variable | HR | P-value | variable | HR | P-value | variable | HR | P-value |
| PCS (PCS1 vs. Others) | 2.75 [1.53-4.95] | <0.001 | PCS (PCS1 vs. Others) | 1.46 [0.99-2.16] | 0.059 | PCS (PCS1 vs. Others) | 2.25 [1.47-3.46] | <0.001 |
| Gleason Score | 1.91 [1.53-2.38] | <0.001 | Gleason Score | 1.5 [1.35-1.66] | <0.001 | Gleason Score | 1.73 [1.51-1.98] | <0.001 |
| PAM50 (LumB vs. Others) | 0.95 [0.57-1.57] | 0.835 | PAM50 (LumB vs. Others) | 1.17 [0.92-1.48] | 0.199 | PAM50 (LumB vs. Others) | 1.21 [0.9-1.64] | 0.207 |
| Decipher Score | 7.42 [2.61-21.06] | <0.001 | Decipher Score | 5.06 [3.15-8.14] | <0.001 | Decipher Score | 8.84 [4.5881-17.05] | <0.001 |

## Slide 5
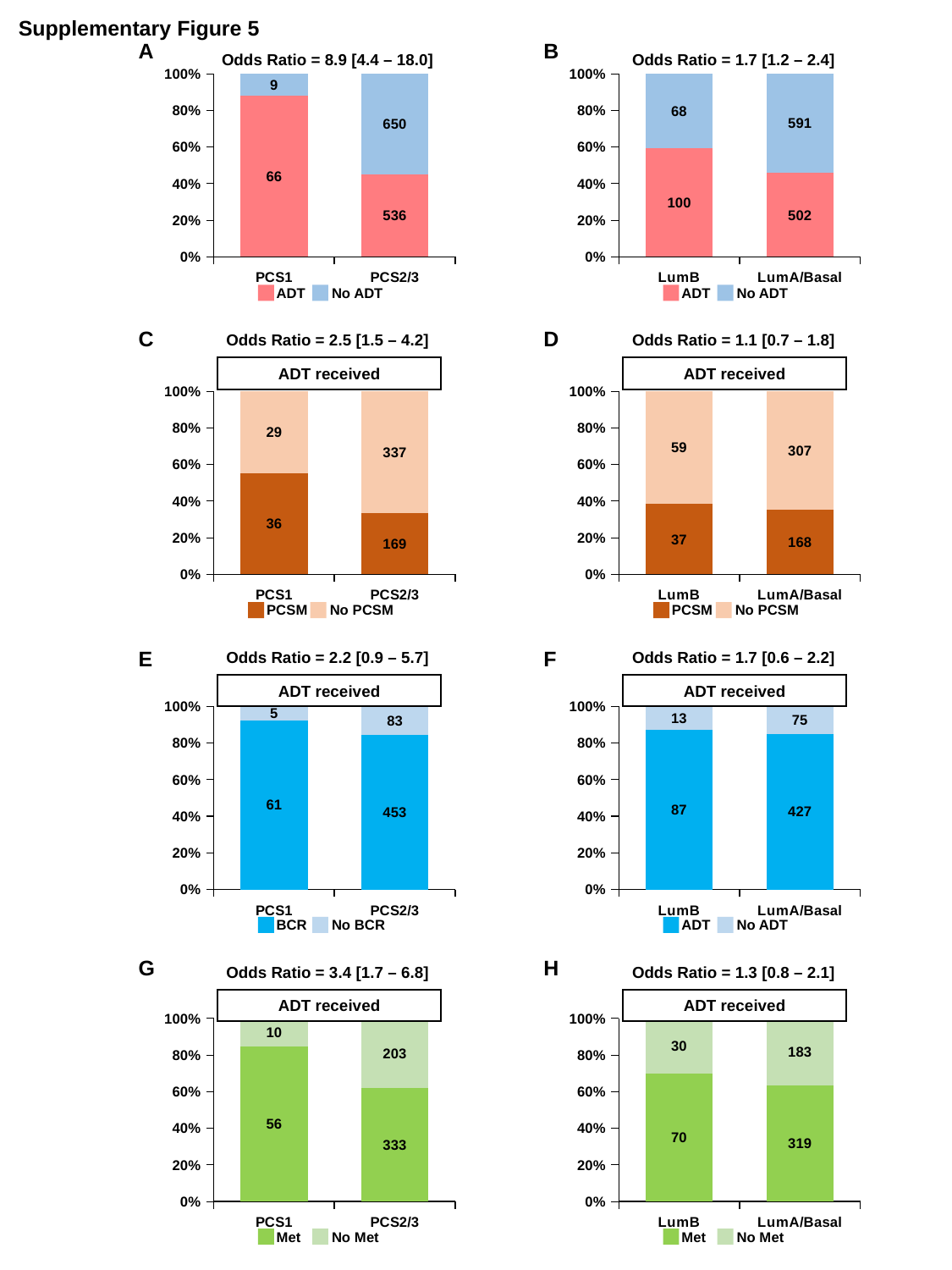

Supplementary Figure 5
A
B
Odds Ratio = 8.9 [4.4 – 18.0]
Odds Ratio = 1.7 [1.2 – 2.4]
### Chart
| Category | | |
|---|---|---|
| PCS1 | 66.0 | 9.0 |
| PCS2/3 | 536.0 | 650.0 |
### Chart
| Category | | |
|---|---|---|
| LumB | 100.0 | 68.0 |
| LumA/Basal | 502.0 | 591.0 |ADT
No ADT
ADT
No ADT
C
D
Odds Ratio = 2.5 [1.5 – 4.2]
Odds Ratio = 1.1 [0.7 – 1.8]
ADT received
ADT received
### Chart
| Category | | |
|---|---|---|
| PCS1 | 36.0 | 29.0 |
| PCS2/3 | 169.0 | 337.0 |
### Chart
| Category | | |
|---|---|---|
| LumB | 37.0 | 59.0 |
| LumA/Basal | 168.0 | 307.0 |PCSM
No PCSM
PCSM
No PCSM
E
F
Odds Ratio = 2.2 [0.9 – 5.7]
Odds Ratio = 1.7 [0.6 – 2.2]
ADT received
ADT received
### Chart
| Category | | |
|---|---|---|
| PCS1 | 61.0 | 5.0 |
| PCS2/3 | 453.0 | 83.0 |
### Chart
| Category | | |
|---|---|---|
| LumB | 87.0 | 13.0 |
| LumA/Basal | 427.0 | 75.0 |BCR
No BCR
ADT
No ADT
G
H
Odds Ratio = 3.4 [1.7 – 6.8]
Odds Ratio = 1.3 [0.8 – 2.1]
ADT received
ADT received
### Chart
| Category | | |
|---|---|---|
| PCS1 | 56.0 | 10.0 |
| PCS2/3 | 333.0 | 203.0 |
### Chart
| Category | | |
|---|---|---|
| LumB | 70.0 | 30.0 |
| LumA/Basal | 319.0 | 183.0 |Met
No Met
Met
No Met

## Slide 6
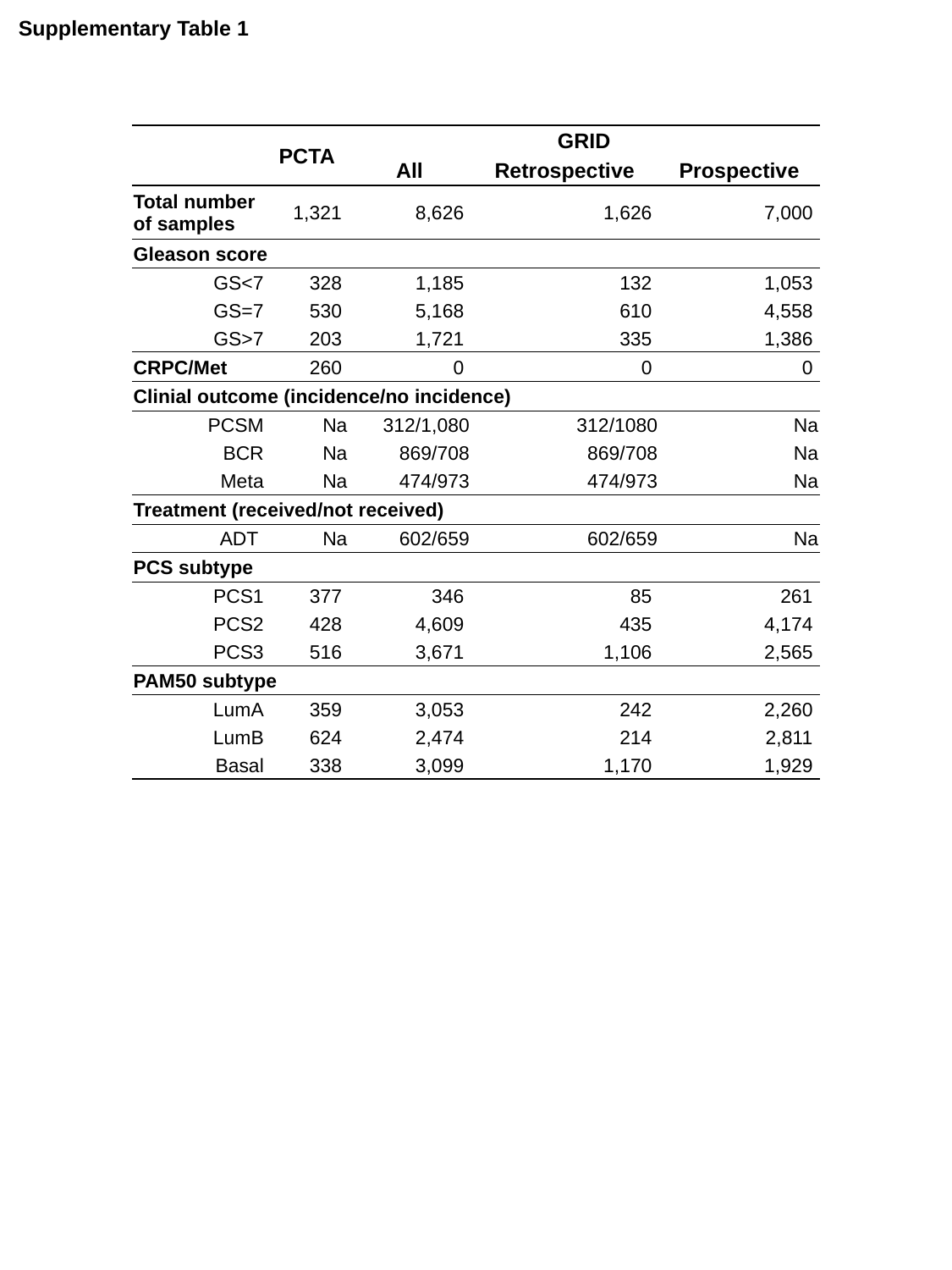

Supplementary Table 1
| | PCTA | GRID | | |
| --- | --- | --- | --- | --- |
| | | All | Retrospective | Prospective |
| Total number of samples | 1,321 | 8,626 | 1,626 | 7,000 |
| Gleason score | | | | |
| GS<7 | 328 | 1,185 | 132 | 1,053 |
| GS=7 | 530 | 5,168 | 610 | 4,558 |
| GS>7 | 203 | 1,721 | 335 | 1,386 |
| CRPC/Met | 260 | 0 | 0 | 0 |
| Clinial outcome (incidence/no incidence) | | | | |
| PCSM | Na | 312/1,080 | 312/1080 | Na |
| BCR | Na | 869/708 | 869/708 | Na |
| Meta | Na | 474/973 | 474/973 | Na |
| Treatment (received/not received) | | | | |
| ADT | Na | 602/659 | 602/659 | Na |
| PCS subtype | | | | |
| PCS1 | 377 | 346 | 85 | 261 |
| PCS2 | 428 | 4,609 | 435 | 4,174 |
| PCS3 | 516 | 3,671 | 1,106 | 2,565 |
| PAM50 subtype | | | | |
| LumA | 359 | 3,053 | 242 | 2,260 |
| LumB | 624 | 2,474 | 214 | 2,811 |
| Basal | 338 | 3,099 | 1,170 | 1,929 |

## Slide 7
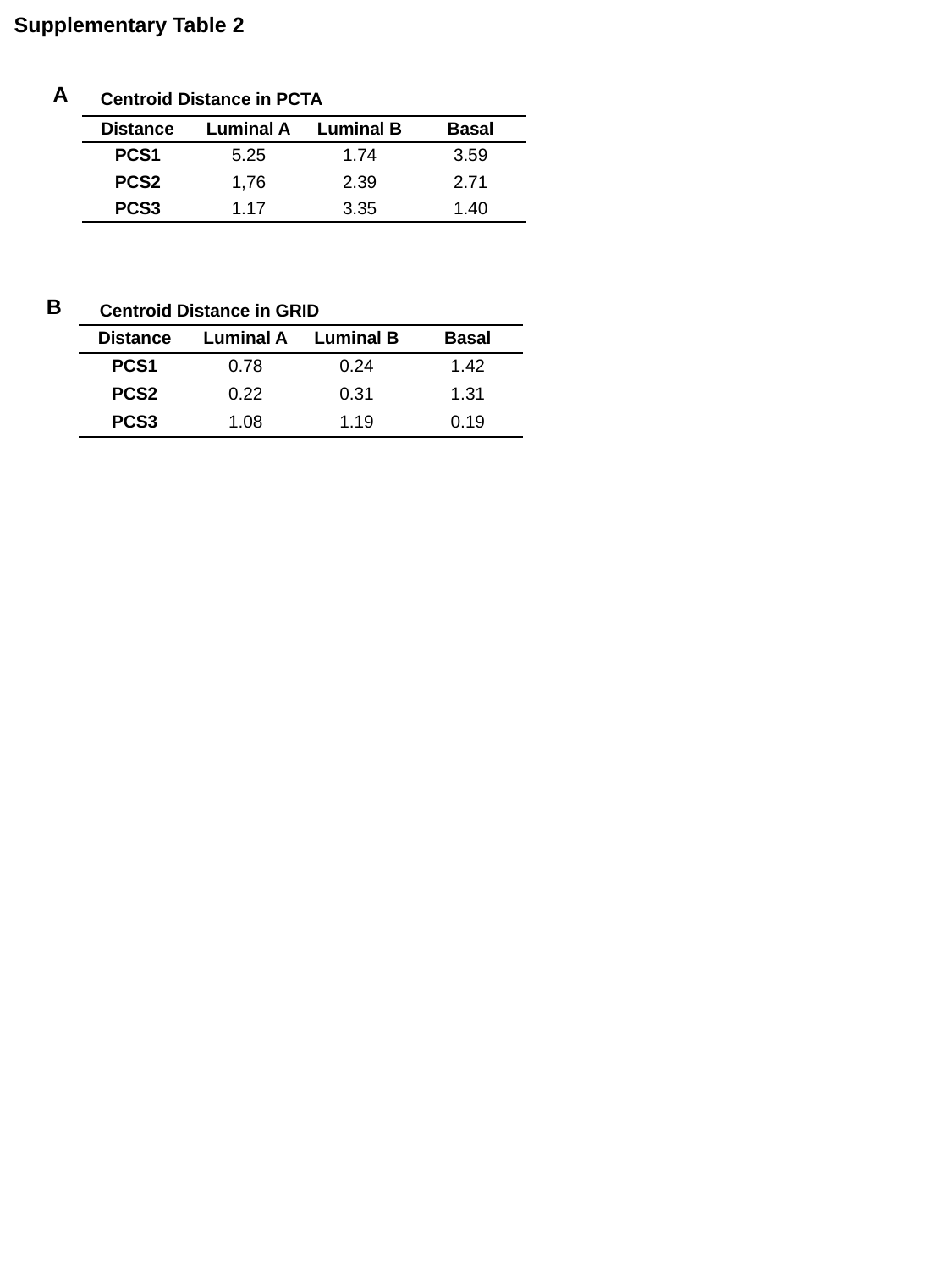

Supplementary Table 2
A
Centroid Distance in PCTA
| Distance | Luminal A | Luminal B | Basal |
| --- | --- | --- | --- |
| PCS1 | 5.25 | 1.74 | 3.59 |
| PCS2 | 1,76 | 2.39 | 2.71 |
| PCS3 | 1.17 | 3.35 | 1.40 |
B
Centroid Distance in GRID
| Distance | Luminal A | Luminal B | Basal |
| --- | --- | --- | --- |
| PCS1 | 0.78 | 0.24 | 1.42 |
| PCS2 | 0.22 | 0.31 | 1.31 |
| PCS3 | 1.08 | 1.19 | 0.19 |

## Slide 8
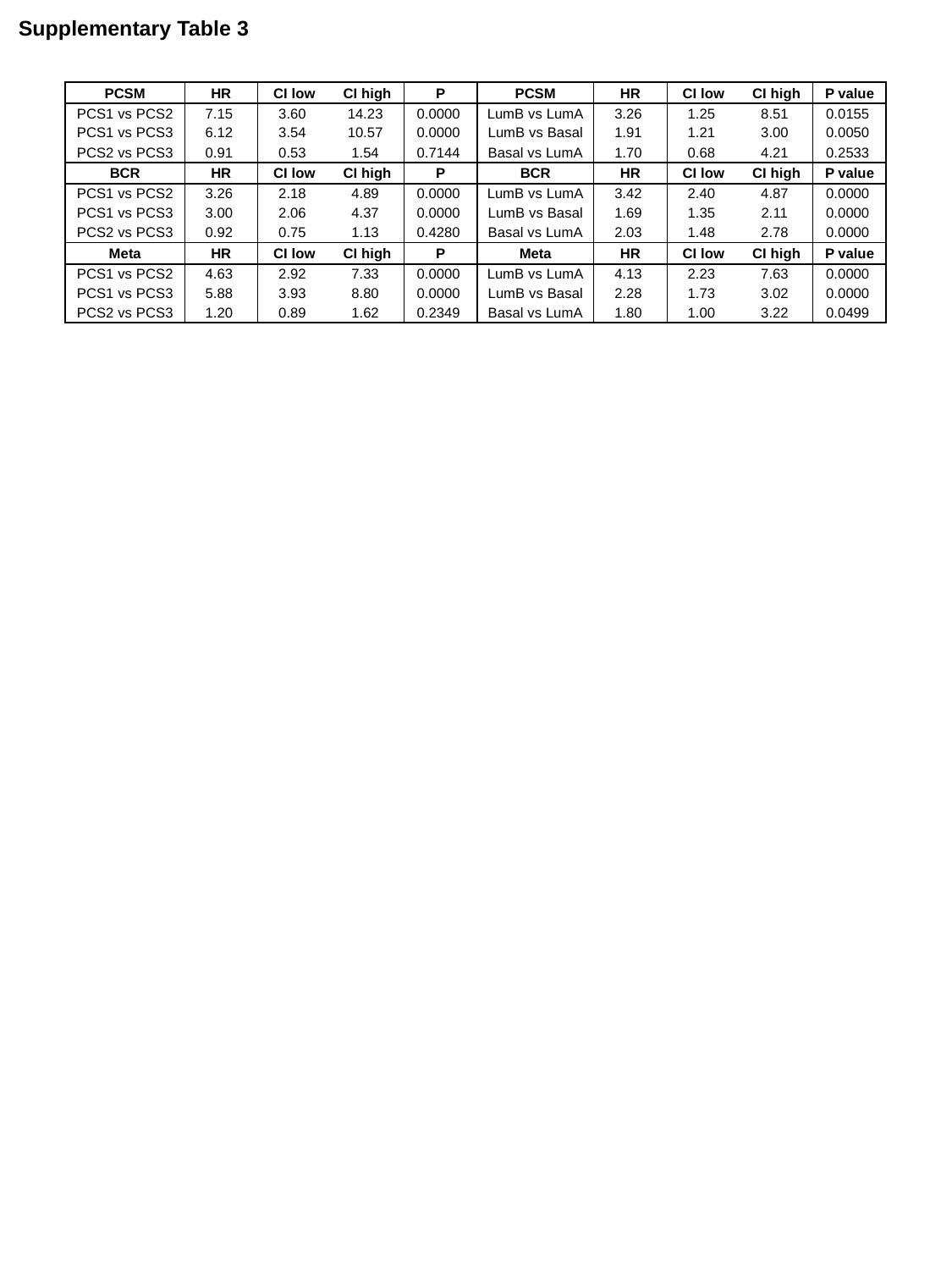

Supplementary Table 3
| PCSM | HR | CI low | CI high | P | PCSM | HR | CI low | CI high | P value |
| --- | --- | --- | --- | --- | --- | --- | --- | --- | --- |
| PCS1 vs PCS2 | 7.15 | 3.60 | 14.23 | 0.0000 | LumB vs LumA | 3.26 | 1.25 | 8.51 | 0.0155 |
| PCS1 vs PCS3 | 6.12 | 3.54 | 10.57 | 0.0000 | LumB vs Basal | 1.91 | 1.21 | 3.00 | 0.0050 |
| PCS2 vs PCS3 | 0.91 | 0.53 | 1.54 | 0.7144 | Basal vs LumA | 1.70 | 0.68 | 4.21 | 0.2533 |
| BCR | HR | CI low | CI high | P | BCR | HR | CI low | CI high | P value |
| PCS1 vs PCS2 | 3.26 | 2.18 | 4.89 | 0.0000 | LumB vs LumA | 3.42 | 2.40 | 4.87 | 0.0000 |
| PCS1 vs PCS3 | 3.00 | 2.06 | 4.37 | 0.0000 | LumB vs Basal | 1.69 | 1.35 | 2.11 | 0.0000 |
| PCS2 vs PCS3 | 0.92 | 0.75 | 1.13 | 0.4280 | Basal vs LumA | 2.03 | 1.48 | 2.78 | 0.0000 |
| Meta | HR | CI low | CI high | P | Meta | HR | CI low | CI high | P value |
| PCS1 vs PCS2 | 4.63 | 2.92 | 7.33 | 0.0000 | LumB vs LumA | 4.13 | 2.23 | 7.63 | 0.0000 |
| PCS1 vs PCS3 | 5.88 | 3.93 | 8.80 | 0.0000 | LumB vs Basal | 2.28 | 1.73 | 3.02 | 0.0000 |
| PCS2 vs PCS3 | 1.20 | 0.89 | 1.62 | 0.2349 | Basal vs LumA | 1.80 | 1.00 | 3.22 | 0.0499 |
